# Discrimination of Pakistani Fountain Pen Inks by Gas Chromatography-Mass Spectrometry (GC-MS)

**DOI:** 10.1155/2022/7186625

**Published:** 2022-03-30

**Authors:** Mehwish Sharif, Muhammad Irfan Jalees, Syed Azhar Ali Shah Tirmazi, Muhammad Makshoof Athar, Arjumand Iqbal Durrani, Madeeha Batool

**Affiliations:** ^1^School of Chemistry, University of the Punjab, Lahore 54590, Pakistan; ^2^Institute of Environmental Engineering and Research, University of Engineering and Technology, Lahore 54890, Pakistan; ^3^Department of Chemistry, University of Engineering and Technology, Lahore 54890, Pakistan

## Abstract

In developing countries, the chances of fraud in written documents are comparatively high. Therefore, comparison of fountain pen inks is especially imperative in examination of forensic questioned documents. We have investigated the use of the gas chromatography-mass spectrometry technique in profiling and discrimination of fountain pen ink used in Pakistan for forensic purpose. The main purpose of this study was to discriminate different Pakistani fountain pen inks. The datum for Pakistani inks of fountain pen is not obtainable. In this research study, blue, black, and green colors fountain pen inks commercially used in Pakistan have been extracted from paper using micropunch and then investigated using the gas chromatography-mass spectrometry technique. Gas chromatography-mass spectrometry (GC-MS) was used to differentiate various brands of different colors of fountain pen inks based on their chemical composition. Molecular ion peaks for different components were obtained, and components were identified on the basis of detected ions. Results have been calculated and compared in terms of discriminating power (D.P.). The D.P. for blue, black, and green inks of fountain pen was 1.0 by using the gas chromatography-mass spectrometry technique.

## 1. Introduction

The fountain pen inks are composed of complex mixture of chemical compounds in common with color imparting products (for example, acid dyes, direct dyes or basic dyes, iron (II) sulfate, and colored pigments of inorganic or organic nature) and additives (for example, surfactants, antioxidants, adjusters for viscosity, glycol, glycerol, and resins) [1]. Compared to other additives of fountain pen ink, dye components are sensitive to light and degrade gradually in normal environmental conditions [2]. Therefore, the analysis of the colorants of dye can give an important information about aging of ink [3, 4].

Components of inks have been categorized with respect to their roles in the matrix of ink. The liquid components are called as vehicles and comprise materials, for example, solvents, oil, and resins that help in ink quality of flow, mechanism of drying, time required for drying, polarity, and its cost. Color substance can be put in as mixture of dyes and pigments, dyes are differentiated by their solubility in the vehicle, while pigments are added as very delicately ground dispersants that are not solubilized. Other components such as waxes that control hardness and flexibility of ink, plasticizers that reduce brittleness of ink and improve stability of ink film, driers that are inorganic salts control drying characteristics, surfactants that change the surface tension of ink and affect its wetting ability, and other miscellaneous materials may also be added that have their particular liabilities [5].

Regarding comparison and identification of inks of fountain pen, there are few studies [6, 7]. Fountain pen has been a popular instrument of writing for above 10 decades and is commonly used to sign official documents in our civilization, for instance, indemnity claims, last wills, tax returns, etc. Illegal cases relating to inaccurate or rewritten documents frequently come across for forensic examination [8].

The capability for differentiation of different inks is of great concern in forensic science as it helps in assessment of the genuineness of a disputed document [9]. Analysis of fountain pen ink on disputed documents is frequently used for ascertaining whether a manuscript is genuine or fabricated, whether entries of writing come from alike ink's composition. For this purpose, the identification of the specific composition of the ink is required. Various analytical methods have been used for the purpose of examination of ink's composition. Various nondestructive techniques such as the video spectral comparator, Raman spectroscopy [10–15], luminescence spectroscopy [16], and destructive techniques such as liquid chromatography [17, 18], capillary zone electrophoresis [19, 20], gas chromatography [21], mass spectrometry [22–24], and gas chromatographymass spectrometry (GC-MS) [25, 26] have been reported to analyse the ink samples. Although gas chromatography-mass spectrometry (GC-MS) is a destructive technique, it has the advantage of enormous adaptability recognition to sensitivity for forensic inks. GC-MS is a well-proven analytical technique for determining the qualitative composition of multicomponent systems such as inks, and therefore, chemical analysis by GC-MS helps to characterize ink-binder resins and solvents [27].

Mass spectra of inks are generally attributed to inorganic substances and dyes used in inks. These dyes and thermally labile persistent components of inks normally generate their mass spectrum as protonated molecules [M + H]^+^ [28]. A research showed that various factors such as composition of ink dyes, type of paper from which the ink was extracted without exposing to light, and natural aging of the ink on document have no effect on mass spectrum [23]. This study aims to investigate and discriminate the composition of different fountain pens used in Pakistan. The study is novel because there are limited studies describing the gas chromatography-mass spectrometry method for forensic discrimination of fountain pen inks, and no literature reference is found for composition of Pakistani fountain pen inks. It is expected that this study will be helpful in the discrimination of samples for fountain pen inks. This data will help in examining alteration and counterfeiting in documents in forensic science using the gas chromatography-mass spectrometry technique.

## 2. Materials and Methods

### 2.1. Sample Preparation

Total eleven ink pots of fountain pen inks commercially available in Pakistan have been obtained (Table 1). With these inks in fountain pen, a straight line has been drawn on A4 size AA paper. The ink writing was left for drying for 20–30 minutes. After drying, microplugs were punched with the help of Harris Aluminum Micro Punch (1 mm). The punched ink sample was then transferred to a preweighted sample vial containing reagent grade water (100 *μ*L). 30 microplugs of paper containing ink were dissolved in 100 *μ*L reagent grade water. These microvials were then sonicated for 10 mins for the extraction of ink from paper punch. The water was then evaporated by passing nitrogen gas through the vials. The dry mass was then dissolved in cyclohexane for gas chromatography-mass spectrometry analysis. For studying the reproducibility of results, six different extractions were made using different writing microplugs of the same ink in separate sample vials. Blank paper sample is also prepared to check if the blank paper contributes any peak in the mass spectrum of pen ink.

### 2.2. Analysis of Fountain Pen Inks by Gas Chromatography-Mass Spectrometry

Gas chromatography-mass spectrometry analysis was performed on the Shimadzu GC-MS QP2010 equipped with a HP-5 column (30 m × 0.32 mm × 0.25 *μ*L) cross-linked with 5% phenyl-methylpolysiloxane. Samples were injected into the gas chromatogram. Helium was used as the carrier gas. The injector temperature was set to 260°C, and the flow rate was 1.2 mL/min. The oven temperature programmed started at 50°C for 1 min and increased at 10°C/min to 200°C with a hold time of 10 minutes. Total run time is 30 minutes. The detector was programmed to scan compounds ranging from 28 to 500 atomic mass units (amu), with ionization energy of 70 ev. LabSolution software is used to process the data and identify the eluted peaks using the National Institute of Standards and Technology (NIST) library database mass spectral search program (Tables 2–4). Relative abundance has been calculated for every peak. During comparison, certain filters such as matched peaks and difference peaks are used.

## 3. Results and Discussion

### 3.1. Identification and % Composition of Inks

**Figure 1:**
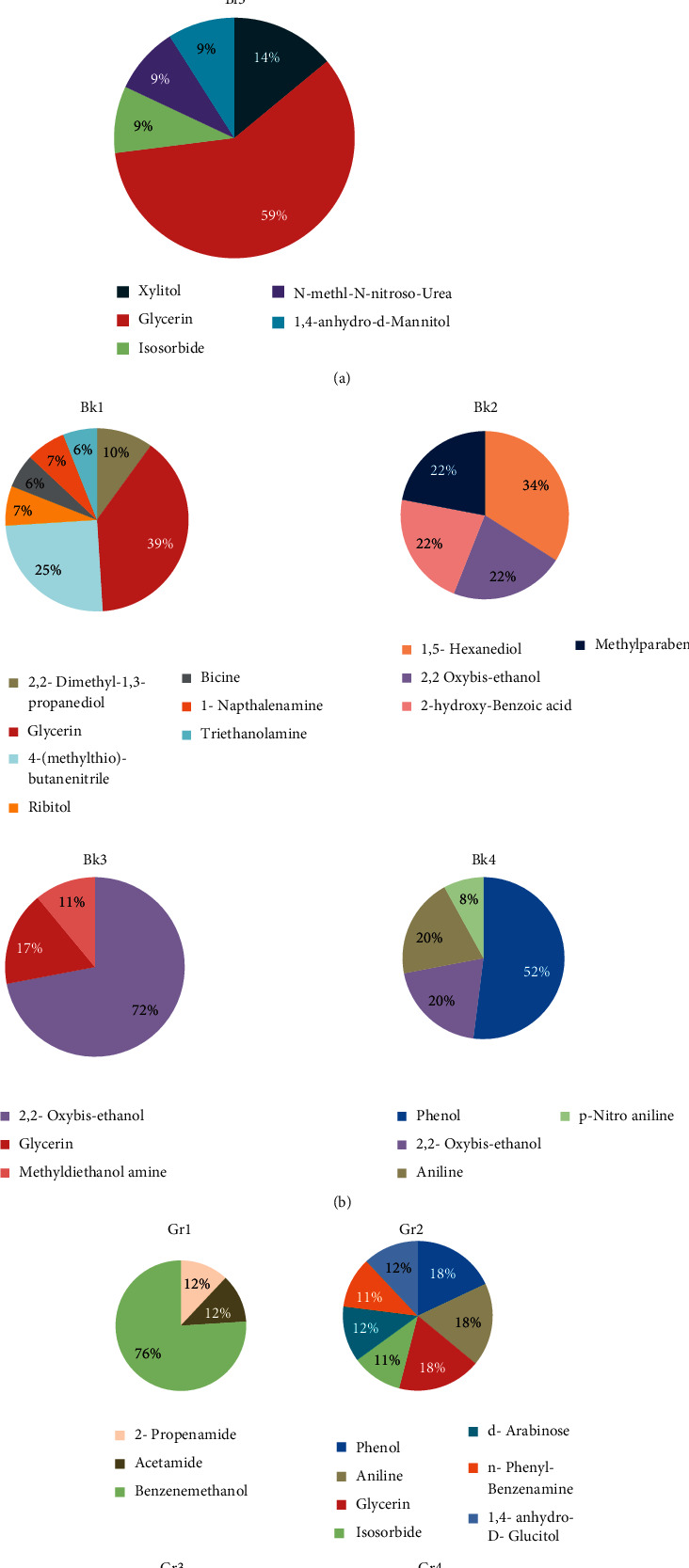
Pie chart representation of (a) blue fountain pen ink samples Bl1, Bl2, and Bl3, (b) black fountain pen ink samples Bk1, Bk2, Bk3, and Bk4, and (c) green fountain pen ink samples Gr1, Gr2, Gr3, and Gr4.

Composition of fountain pen inks used in this study can be explained with the help of gas chromatography-mass spectrometry results. Results showed that ink samples were composed of variety of components; however, their percentage varied from sample to sample, as shown in Figure 1.

**Figure 2:**
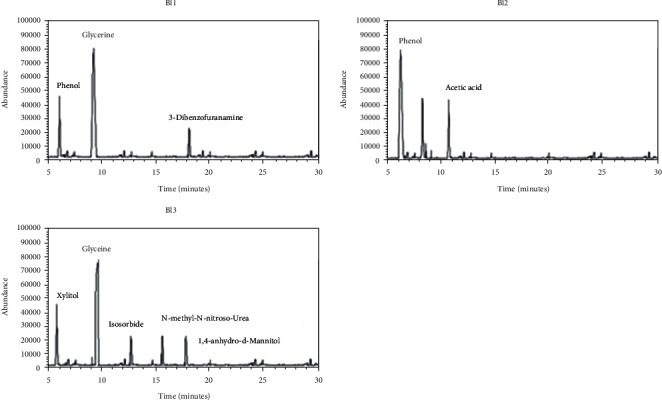
Total ion chromatogram (TIC) obtained from GC-MS analysis of blue fountain pen ink samples Bl1, Bl2, and Bl3.

Chemical analysis of blue inks of fountain pen (Bl1–Bl3) by GC-MS indicated the presence of phenol in Bl1 (16%) and Bl2 (68%) (Figure 2 and Table 2) and its absence in Bl3, whereas glycerin was found in Bl1 (73%) and Bl3 (59%). 3-Dibenzofuran amine (11%) was found only in Bl1. Similarly, acetic acid (16%) was found in Bl2. Isosorbide (9%), N-methyl-N-nitroso-urea (9%), and 1,4-anhydro-d-mannitol (9%) were found in Bl3.

**Figure 3:**
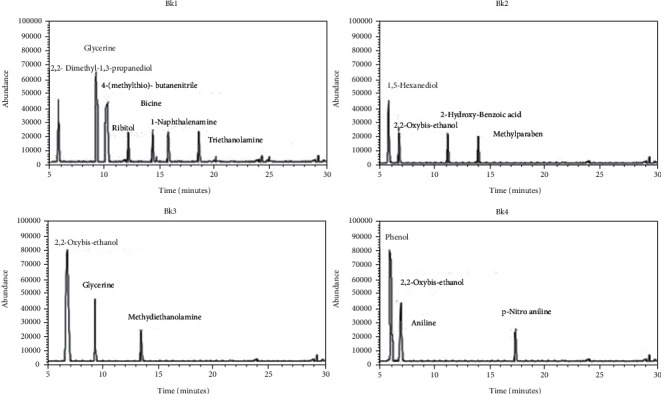
Total ion chromatogram (TIC) obtained from GC-MS analysis of black fountain pen ink samples Bk1, Bk2, Bk3, and Bk4.

Among black fountain pen inks, phenol (52%) was found in Bk4, while absent in rest of other samples, as shown in Figure 3 and Table 3. Different percentages of 2,2-dimethyl-1,3-propanediol (10%), 4-(methylthio) butane nitrile (25%), ribitol (7%), triethanolamine (6%), bicine (6%), and 1-naphthalene amine (7%) were found in Bk1, while not found in the other samples of fountain pen inks of black color. 2,2-Oxy bis-ethanol (22%) was found in Bk2, 72% in Bk3, and 20% in Bk4. Glycerin was found 39% in Bk1 and 17% in Bk3. 1,5-Hexanediol (34%), 2-hydroxy-benzoic acid (22%) and methylparaben (22%) were found only in Bk2. Methyl diethanol amine (11%) was found only in Bk3. Aniline (20%) and p-nitro aniline (8%) were found only in Bk4.

**Figure 4:**
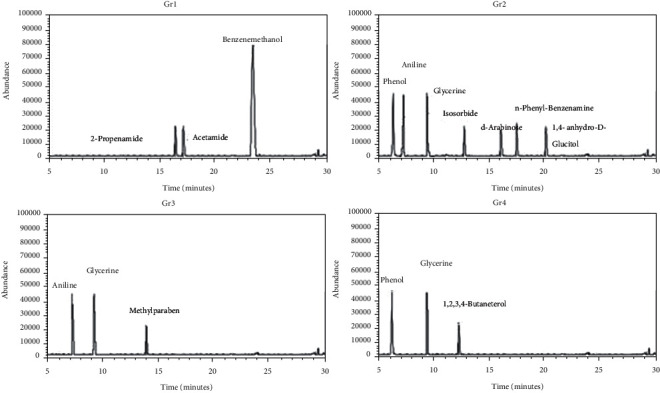
Total ion chromatogram (TIC) obtained from GC-MS analysis of green fountain pen ink samples Gr1, Gr2, Gr3, and Gr4.

Analysis of green fountain pen inks (Figure 4 and Table 4) has shown that phenol was found in both Gr2 (18%) and Gr4 (37%); however, difference in percentage of phenol in both the samples made these samples distinguishable. Glycerin was found in Gr2 (18%), Gr3 (38%), and Gr4 (38%). Methylparaben (25%) was found in Gr3 only. Isosorbide (11%) was found only in Gr2. 2-Propenamide (12%), acetamide (12%), and benzene methanol (76%) were found only in Gr1. Aniline was found only in Gr2 (18%) and Gr3 (37%), while not found in the other samples of green fountain pen inks. n-Phenyl-benzenamine (11%), d-arabinose (12%) and 1,4-anhydro-D-D-glucitol (12%) were found only in Gr2, and 1,2,3,4-butanetetrol (25%) was found only in Gr4. Therefore, it was evident that all fountain pen ink samples showed different composition using gas chromatography-mass spectrometry.

### 3.2. Discriminating Power

The term discriminating power (D.P.) for an item expresses how quickly the change happens from low probability to high probability of an accurate response. A greatly discriminating article provide superior end results with least chances for ambiguities. Comparison of two articles can be completed in a direct way by using discriminating power to determine which can superiorly calculate a specific characteristic [29]. Discriminating power is computed according to the following formula accessible in the literature [30].

(1) $$\begin{array}{c} \text{D}.\text{P}=\frac{\text{number of discriminating sample pair}}{\text{number of possible sample pair}}. \end{array}$$

All samples of blue, black, and green fountain pen inks have been separated, and discriminating power calculated by abovementioned formula is 1.0 for fountain pen inks of blue, black, and green colors using GC-MS as given in Table 5, which indicates that all samples of blue, black, and green fountain pen inks have been distinguished 100%.

The available literature values of discriminating powers for fountain pen inks of blue, black, and green colors are 0.73, 0.87, and 0.83 for blue, black, and green colored fountain pen inks, respectively, by using ultraviolet/visible spectroscopy [31]. Discriminating powers were reported as 0.80, 1.0, and 1.0 for blue, black, and green colored fountain pen inks, correspondingly, by TLC (thin layer chromatography) [31]. Discriminating power was described as 0.73, 0.80, and 0.5 for fountain pen inks of blue, black, and green color, correspondingly, with the use of Fourier transform infrared spectroscopy (FTIR spectroscopy) [31]. However, no literature reference for discriminating power of fountain pen inks by using gas chromatography-mass spectrometry has been found.

Previous study for fountain pen inks of same samples by Sharif et al. [31] reported successful discrimination of samples by combination of techniques. By using TLC for blue inks of fountain pen, BL1 and BL3 cannot be discriminated but discriminated by using ultraviolet/visible spectroscopy and Fourier transform infrared spectroscopy. Black fountain pen ink samples, BK1 and BK3, have not been discriminated by ultraviolet/visible spectroscopy but discriminated by TLC and FTIR spectroscopies. Similarly, green color fountain pen ink samples, GN1 and GN3, have not been discriminated by ultraviolet/visible spectroscopy but have been discriminated by TLC and FTIR spectroscopies. GN1, GN2; GN1, GN4; and GN2, GN4 have not discriminated by Fourier transform infrared spectroscopy but have been discriminated by ultraviolet/visible spectroscopy and thin layer chromatography.

## 4. Conclusion

With the help of the gas chromatography-mass spectrometry technique, all samples of blue, black, and green fountain pen inks can be differentiated as the chemical constituents show differentiation. The discriminating power for fountain pen inks of blue, black, and green color by the gas chromatography-mass spectrometry technique is 1.0, as all sample pairs have been discriminated successfully. These results indicate that gas chromatography-mass spectrometry is the most efficient separation technique for fountain pen inks, as it can properly discriminate fountain pen ink samples. Therefore, it is suggested that gas chromatography-mass spectrometry should be used wherever to deal with fraud cases based on misuse of inks.

In our current study, all samples have been discriminated by using one technique, i.e., gas chromatography-mass spectrometry. Therefore, gas chromatography-mass spectrometry is found to be the most efficient technique as compared to UV/visible spectroscopy, TLC, and FTIR spectroscopy.

Supplementary learn for determination of content of metals in inks of blue, black, and green color fountain pens has been suggested for improved discrimination.

## Figures and Tables

**Table 1:** Samples of fountain pen inks of different colors and brands with sample codes for gas chromatography-mass spectrometry.

Sr. no.	Blue		Black		Green	
	Ink brand	Sample code	Ink brand	Sample code	Ink brand	Sample code
1	Dollar (blue)	Bl1	Sheaffer (black)	Bk1	Pelikan (turquoise)	Gr1
2	Decent (blue)	Bl2	Parker Quink (black)	Bk2	Pelikan (green)	Gr2
3	Pelikan (blue)	Bl3	Pelikan (black)	Bk3	Parker Quink (green)	Gr3
4	—	—	Dollar (black)	Bk4	Dollar (green)	Gr4

**Table 2:** Compounds identified by GC-MS for blue fountain pen inks samples: Bl1, Bl2, and Bl3.

Ink code	Peak identification	Retention time (min)	Chemical formula	Relative abundance
Bl1	Phenol	6.370	(C_6_H_6_O)	5.42
	Glycerin	9.561	(C_3_H_8_O_3_)	24.02
	3-Dibenzofuranamine	18.166	(C_12_H_9_NO)	3.47

Bl2	Phenol	6.348	(C_6_H_6_O)	23.23
	Acetic acid	10.770	(C_2_H_4_O_2_)	5.40

Bl3	Xylitol	5.941	(C_5_H_12_O_5_)	5.22
	Glycerin	9.55	(C_3_H_8_O_3_)	22.50
	Isosorbide	12.630	(C_6_H_10_O_4_)	3.43
	N-Methyl-N-nitroso-urea	15.674	(C_2_H_5_N_3_O_2_)	3.62
	1,4-Anhydro-d-mannitol	17.846	(C_6_H_12_O_5_)	3.43

**Table 3:** Compounds identified by GC-MS for black fountain pen inks samples: Bk1, Bk2, Bk3, and Bk4.

Ink code	Compound	Retention time (min)	Chemical formula	Relative abundance
Bk1	2,2-Dimethyl-1,3-propanediol	5.841	(C_5_H_12_O_2_)	5.23
	Glycerin	9.579	(C_3_H_8_O_3_)	20.82
	4-(Methylthio)-butanenitrile	10.398	(C_5_H_9_NS)	13.14
	Ribitol	12.101	(C_5_H_12_O_5_)	3.53
	Bicine	14.295	(C_6_H_13_NO_4_)	3.43
	1-Napthalenamine	15.643	(C_10_H_9_N)	3.47
	Triethanolamine	18.474	(C_6_H_15_NO_3_)	3.44

Bk2	1,5-Hexanediol	5.954	(C_6_H_14_O_2_)	6.89
	2,2-Oxybis-ethanol	6.808	(C_4_H_10_O_3_)	4.57
	2-Hydroxy-benzoic acid	11.230	(C_7_H_6_O_3_)	4.60
	Methylparaben	13.909	(C_8_H_8_O_3_)	4.53

Bk3	2,2-Oxybis-ethanol	7. 098	(C_4_H_10_O_3_)	24.61
	Glycerin	9.57	(C_3_H_8_O_3_)	5.74
	Methyldiethanolamine	13.376	(C_5_H_13_NO_2_)	3.75

Bk4	Phenol	6.33	(C_6_H_6_O)	23.84
	2,2-Oxybis-ethanol	7.098	(C_4_H_10_O_3_)	9.05
	Aniline	7.280	(C_6_H_7_N)	9.05
	p-Nitro aniline	17.126	(C_6_H_6_N_2_O_2_)	3.64

**Table 4:** Compounds identified by GC-MS for green fountain pen inks samples: Gr1, Gr2, Gr3, and Gr4.

Ink code	Compounds	Retention time (min)	Chemical formula	Relative abundance
Gr1	2-Propenamide	16.385	(C_3_H_5_NO)	3.84
	Acetamide	16.974	(C_2_H_5_NO)	3.84
	Benzenemethanol	23.351	(C_7_H_8_O)	25.17

Gr2	Phenol	6.377	(C_6_H_6_O)	5.95
	Aniline	7.275	(C_6_H_7_N)	5.98
	Glycerin	9.570	(C_3_H_8_O_3_)	5.95
	Isosorbide	12.847	(C_6_H_10_O_4_)	3.92
	d-Arabinose	16.112	(C_5_H_10_O_5_)	3.94
	n-Phenyl-benzenamine	17.356	(C_12_H_11_ N)	3.92
	1,4-Anhydro-D-glucitol	19.931	(C_6_H_12_O_5_)	3.98

Gr3	Aniline	7.265	(C_6_H_7_N)	6.99
	Glycerin	9.579	(C_3_H_8_O_3_)	7.11
	Methylparaben	13.909	(C_8_H_8_O_3_)	4.60

Gr4	Phenol	6.348	(C_6_H_6_O)	6.99
	Glycerin	9.580	(C_3_H_8_O_3_)	7.03
	1,2,3,4-Butanetetrol	12.171	(C_4_H_10_O_4_)	4.64

**Table 5:** Discriminating power for blue, black, and green fountain pen ink samples using gas chromatography-mass spectrometry.

Color of fountain pen ink	*n* = total no. of samples	Total no. of pairs = *n*(*n*−1)/2	Discriminating pairs (total no.)	Nondiscriminating pairs (total no.)	Discriminating power = no. of discriminating pairs/total no. of pairs
Blue	3	3*∗*2/2 = 3	Bl1, Bl2	0	3/3 = 1.0
			Bl1, Bl3		
			Bl2, Bl3		

Black	4	4*∗*3/2 = 6	Bk1, Bk2	0	6/6 = 1.0
			Bk1, Bk3		
			Bk1, Bk4		
			BK2, BK3		
			BK2, BK4		
			Bk3, Bk4		

Green	4	4*∗*3/2 = 6	Gr1, Gr2	0	6/6 = 1.0
			Gr1, Gr3		
			Gr1, Gr4		
			Gr2, Gr3		
			Gr2, Gr4		
			Gr3, Gr4		

## Data Availability

The data used to support the findings of this study are available from the corresponding author upon request.
